# Activation of Akt–mTORC1 signalling reverts cancer‐dependent muscle wasting

**DOI:** 10.1002/jcsm.12854

**Published:** 2021-11-06

**Authors:** Alessia Geremia, Roberta Sartori, Martina Baraldo, Leonardo Nogara, Valeria Balmaceda, Georgia Ana Dumitras, Stefano Ciciliot, Marco Scalabrin, Hendrik Nolte, Bert Blaauw

**Affiliations:** ^1^ Veneto Institute of Molecular Medicine (VIMM) Padua Italy; ^2^ Department of Biomedical Sciences University of Padua Padua Italy; ^3^ Max Planck Institute for Biology of Ageing Cologne Germany

**Keywords:** Cancer cachexia, mTOR, Raptor, Akt, Muscle growth, Skeletal muscle force

## Abstract

**Background:**

Cancer‐related muscle wasting occurs in most cancer patients. An important regulator of adult muscle mass and function is the Akt–mTORC1 pathway. While Akt–mTORC1 signalling is important for adult muscle homeostasis, it is also a major target of numerous cancer treatments. Which role Akt–mTORC1 signalling plays during cancer cachexia in muscle is currently not known. Here, we aimed to determine how activation or inactivation of the pathway affects skeletal muscle during cancer cachexia.

**Methods:**

We used inducible, muscle‐specific Raptor ko (mTORC1) mice to determine the effect of reduced mTOR signalling during cancer cachexia. On the contrary, in order to understand if skeletal muscles maintain their anabolic capacity and if activation of Akt–mTORC1 signalling can reverse cancer cachexia, we generated mice in which we can inducibly activate Akt specifically in skeletal muscles.

**Results:**

We found that mTORC1 signalling is impaired during cancer cachexia, using the Lewis lung carcinoma and C26 colon cancer model, and is accompanied by a reduction in protein synthesis rates of 57% (*P* < 0.01). Further reduction of mTOR signalling, as seen in Raptor ko animals, leads to a 1.5‐fold increase in autophagic flux (*P* > 0.001), but does not further increase muscle wasting. On the other hand, activation of Akt–mTORC1 signalling in already cachectic animals completely reverses the 15–20% loss in muscle mass and force (*P* < 0.001). Interestingly, Akt activation only in skeletal muscle completely normalizes the transcriptional deregulation observed in cachectic muscle, despite having no effect on tumour size or spleen mass. In addition to stimulating muscle growth, it is also sufficient to prevent the increase in protein degradation normally observed in muscles from tumour‐bearing animals.

**Conclusions:**

Here, we show that activation of Akt–mTORC1 signalling is sufficient to completely revert cancer‐dependent muscle wasting. Intriguingly, these results show that skeletal muscle maintains its anabolic capacities also during cancer cachexia, possibly giving a rationale behind some of the beneficial effects observed in exercise in cancer patients.

## Introduction

Cancer cachexia is a multi‐organ syndrome, which is characterized by a strong loss in body weight. It occurs in 50–80% of cancer patients and is due to a drastic loss in muscle and adipose tissue.^1^ As cancer cachexia leads to a decrease in physical performance and is associated with poor survival (accounting for more than 20% of cancer deaths), it is of major clinical relevance. Furthermore, cachectic patients show lower response rates to chemotherapy and a reduced tolerance to anticancer treatment.^2^ However, despite its clinical importance and the foreseen impact on patients, the pathophysiology of cachexia‐associated muscle wasting is still poorly understood.

One of the major intracellular pathways regulating adult skeletal muscle mass and function is the Akt–mTORC1 pathway.^3^, ^4^ Phosphorylation of the serine/threonine kinase Akt leads to the activation of the kinase complex mTORC1, that is, when the kinase mTOR is complexed with the scaffold protein Raptor. Activation of this pathway is accompanied by an increase in protein synthesis and reduction in protein degradation, leading to the growth of adult skeletal muscle.^5^ On the contrary, prolonged reductions in mTORC1 signalling in muscle fibres can negatively affect muscle homeostasis, by dysregulating autophagy and even leading to impaired muscle innervation.^6^, ^7^ Interestingly, in addition to the well‐established loss in muscle mass, it was recently shown that cancer cachexia is also accompanied by the appearance of fibre denervation,^8^ making it tempting to link cancer cachexia to altered mTORC1 signalling. When examining the activation levels of Akt–mTORC1 signalling in tumour‐bearing animals, different observations have been made. Mice inoculated with Lewis lung carcinoma (LLC) or colon‐26 carcinoma (C26) have shown reductions in mTORC1 signalling.^9^, ^10^ The same was observed in transgenic mice, which spontaneously develop intestinal polyps, leading to a cachectic phenotype between 3 and 6 months of age.^11^ Interestingly, treatments that show an improvement of the cachectic muscle phenotype, like treadmill running or the natural plant product salidroside, are accompanied by a restoration of mTORC1 signalling.^10^, ^12^ These results are somewhat in contrast to another study, which showed that treatment of C26 mice with the mTORC1 inhibitor rapamycin can reduce muscle atrophy, possibly by restoring autophagy to normal levels.^13^

In patients, the link between mTORC1 signalling and cachexia is not that clear; however, there are reports that suggest that the systemic use of mTOR inhibitors can aggravate cancer cachexia. Indeed, long‐term treatment with mTOR inhibitors was accompanied by a loss in muscle mass, while not affecting fat mass or body weight.^14^ Furthermore, patients with metastatic renal cell cancer, which were treated with inhibitors that block mTOR signalling, showed a significant exacerbation in muscle wasting.^15^

Here, we use various transgenic mouse strains to determine the role of Akt–mTORC1 signalling in skeletal muscle during cancer cachexia. We find that loss of Raptor only in muscle fibres does not aggravate cancer‐related muscle wasting. On the contrary, activation of Akt–mTORC1 signalling is sufficient to completely revert muscle wasting, normalize the muscle transcriptome, and prevent protein degradation.

## Materials and methods

### Animal experiments and treatments

Inducible muscle‐specific Raptor knockout mice were generated as described previously.^7^ Briefly, mice expressing the floxed Raptor gene (Raptor^fl/fl^) were crossed with mice carrying the Cre recombinase fused to a mutated oestrogen receptor (ER) domain under the control of human skeletal actin promoter (HSA).^16^ In Raptor ko mice, tamoxifen‐induced Cre LoxP recombination was achieved by intraperitoneal injection of tamoxifen (3 mg) once daily for 1 week in 2‐month‐old mice. C26 cells were injected 3 weeks after the end of tamoxifen treatment. Mice were backcrossed at least for two generations in a BALB/c background. Both male and female mice were used.

Inducible Akt transgenic mice were generated as described previously.^5^, ^17^ Briefly, a transgenic line that expresses the Cre recombinase under a muscle‐specific (myosin light chain 1 fast) promoter was crossed with a second line that expresses Akt1 only after the deletion of a floxed upstream sequence by the Cre recombinase. The Akt coding sequence is fused to a modified ER‐binding domain; Akt phosphorylation and activation are induced only by exogenous treatment of tamoxifen, which binds the ER.

C26 and LLC cells, a gift from Paola Costelli's lab, were cultured in high glucose DMEM (# 41966 Gibco). All culture media were supplemented with 10% foetal bovine serum (Gibco) and Pen/Strep solution (penicillin 100 U/mL and streptomycin 0.1 mg/mL, Gibco). All cell cultures were maintained at 37°C with a humidified atmosphere of 5% CO_2_. Low‐passage cell lines were used. C26 and LLC cell suspensions were injected subcutaneously dorsally in 3‐month‐old mice, BALB‐c or C57BL/6 strain, respectively. Tumour‐bearing mice received 5 × 10^5^ C26 or LLC cells in physiological solution. Control mice were inoculated with physiological solution. In all experimental models of cancer cachexia, mice were treated until they reached the experimental endpoint, determined by ethical criteria (loss of 20% initial body mass). C26 mice were sacrificed when they reached −20% of body weight, generally 14 days after inoculation; LLC mice were sacrificed after 30 days, at −20% of body weight or at reaching of a humane endpoint. In Akt mice, C26 cells were injected in 3‐month‐old mice, and when they lost 10–12% of initial body weight, 1 mg of tamoxifen was injected intraperitoneally for 5 days to activate Akt. Akt mice were sacrificed when they regained body weight after tamoxifen treatment. Control mice received tamoxifen as well, and they were sacrificed when they lost 20% of body weight. A detailed scheme for the mouse models used is provide in Supporting Information, *Figure* S1.

Analysis of body composition in mice was performed by quantitative magnetic resonance using an EchoMRI™‐100 (EchoMRI LLC, TX, USA) without the use of anaesthesia.

Colchicine was used to monitor autophagic flux as described previously.^7^ Wt and Raptor ko, tumour‐bearing mice and sham‐treated controls were treated with 0.4 mg/kg of colchicine or vehicle by intraperitoneal injection. The treatment was repeated twice, 24 and 12 h prior to muscle collection. Puromycin was injected intraperitoneally with 0.04 μmol/g puromycin exactly 30 min before removal of muscles as described previously.^18^

Experimental protocols were reviewed and approved by the local Animal Care Committee, University of Padua.

### *In vivo* muscle force measurement

Gastrocnemius muscle force was measured in living mice as previously described.^19^ Briefly, animals were anaesthetized, and muscle contractile performance was measured *in vivo* using a 305B muscle lever system (Aurora Scientific Inc.). Contraction was elicited by electrical stimulation of the sciatic nerve. Common peroneal nerve was cut, and the torque developed during isometric contractions was measured by stepwise increasing stimulation frequency, with pauses of at least 30 s between stimuli to avoid fatigue. Duration of the stimulation trains was 600 ms. Force was normalized to the muscle mass as an estimate of specific force. Animals were then sacrificed by cervical dislocation, and muscles were dissected, weighted, and frozen.

### Antibodies and western blotting

We used the following antibodies for western blotting: pAKT (S473; Ref. 4060), pS6 (S240/244; Ref. 5364), S6 (Ref. 2217), AKT (Ref. 9272), p4E‐BP1 (Thr37/46; Ref. 2855), 4E‐BP1 (Ref. 9644), RAPTOR (Ref. 2280), and mTOR (Ref. 2983) from Cell Signaling; puromycin (clone 12D10; Ref. MABE343) and Lys48 (Ref. 04‐263) from Millipore; GAPDH (Ref. 8245) from Abcam; actin (Ref. 56459) from Santa Cruz; and LC3 (Ref. L7543) and p62 (Ref. P0067) from Sigma. For immunofluorescence, NCAM was from Millipore, and 488‐conjugate wheat germ agglutinin (Ref. W11261) was from Invitrogen. For lysate preparation, cryosections of 20 μm of gastrocnemius (GC) muscles were lysed in 100 μL of a buffer containing 50 mM Tris pH 7.5, 150 mM NaCl, 10 mM MgCl_2_, 0.5 mM DTT, 1 mM EDTA, 10% glycerol, 2% SDS, 1% Triton X‐100, Roche Complete Protease Inhibitor Cocktail, and Roche Phospho‐Stop Phosphatase Inhibitor Cocktail. To detect Lys63 and Lys48 polyubiquitin chain content, samples were also added to two proteasome inhibitors: MG132 (Tocris Bioscience) and NEM (Sigma‐Aldrich). Lysates were incubated at 70°C for 10 min and centrifuged at 16 000 *g* for 15 min at 4°C. Concentration of supernatant protein was, then, measured using BCA Protein Assay Kit (Pierce) following manufacturer's instructions.

### Gene expression analysis

Total RNA was extracted from muscles using TRIzol (Invitrogen). Complementary DNA was generated from 0.4 μg of RNA reverse transcribed with SuperScript III Reverse Transcriptase (Invitrogen). cDNA samples were then amplified on the 7900HT Fast Real‐Time PCR System (Applied Biosystems) using the Power SYBR Green RT‐PCR Kit (Applied Biosystems). All data were normalized to HPRT and to GAPDH expression.

### Mito‐Keima experiment

Analysis was performed as described previously.^7^ Briefly, electroporation was performed on FDB muscles from wild‐type and knockout animals, C26, and shams. FDB muscles were collected in 1% P/S Dulbecco's modified Eagle's medium, dissociated, and plated on glass coverslips coated with 10% Matrigel in Tyrode's salt solution (pH 7.4). Mitochondria‐targeted mito‐Keima plasmid (mito‐Keima, MBL International) was used to monitor mitophagy in transfected FSB single fibres. Fluorescence of mito‐Keima was imaged in two channels via two sequential excitations (458 nm, green; 561 nm, red) and using a 570–796 nm emission range. The level of mitophagy was defined as the total number of red pixels divided by the total number of all pixels.

### Periodic acid–Schiff quantification

For PAS quantification, images of four TA sections per group were acquired using the same exposure settings on a Leica DM6B. Images were acquired using LAS X software and analysed with Fiji. In brief, pictures have been converted to 8 bits; surrounding background and imperfections have been excluded from the analysis by converting their value to full black. An increasing threshold has been run through the picture, and the selected pixel has been measured for mean intensity and area, obtaining a distribution profile for each picture. Measure values are expressed in terms of percentage of area for each section above a common threshold, which has been determined as the mean intensity of the control group pictures (wt sham).^20^, ^21^

### RNA‐Seq

Total RNA was isolated from GC muscle using TRIzol reagent (Life Technologies) according to the manufacturer's instructions (*n* = 3 per group). Total RNA was submitted to CRIBI—Biology Department, University of Padua. RNA was checked for purity and integrity in a Bioanalyzer device (Agilent Technologies, Inc., Santa Clara, CA, USA), and Quant Seq 3′ mRNA‐seq Library Prep kit (Lexogen) is used for library construction. Sequencing is performed on NextSeq 500 Illumina instrument to produce 5 million reads (75 bp SE) for sample.

### Statistical analysis

Statistical tests [Student's *t*‐test and two‐way analysis of variance (ANOVA)] were used as described in the figure legends. For all graphs, data are presented as means ± SEM. The *P* values are reported in the figure legends, and *P* value <0.05 was considered statistically significant. Statistical analyses were performed using GraphPad PRISM 9.0a (GraphPad, La Jolla, CA, USA). For RNA‐Seq data analysis, we applied a two‐sided *t*‐test as well as a two‐way ANOVA in the Perseus software,^22^ and for pairwise comparisons, a permutation‐based approach to correct for multiple testing. For visualization, the software Instant Clue was utilized^23^; hierarchical clustering was performed using the Euclidean distance and complete method. The principal component analysis was performed after scaling the data to unit variance and mean zero.

## Results

### Cancer cachexia is associated with reduced Akt–mTORC1 signalling

**Figure 1:**
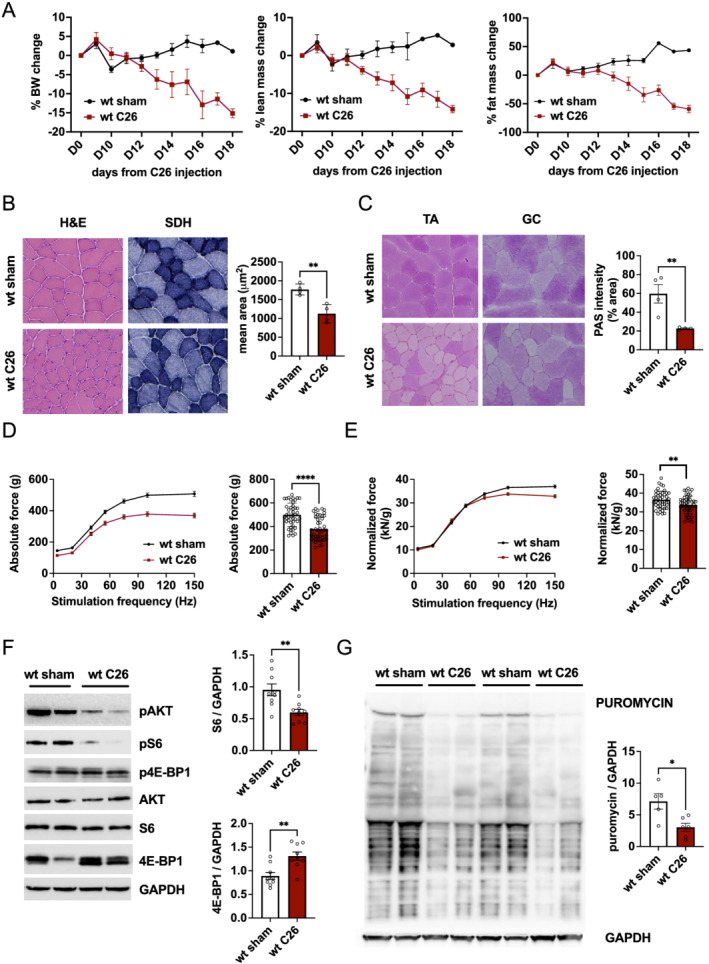
C26 cancer cachexia model is accompanied by a decrease in mTORC1 signalling and protein synthesis in skeletal muscle. (A) Progressive decline in body weight and lean and fat mass in C26 tumour‐bearing mice and sham controls (*n* = 10 mice per group). (B) Representative haematoxylin & eosin (H&E) and succinate dehydrogenase (SDH) staining in cryosections of tibialis anterior (TA) muscle from cachectic and control mice; cross‐sectional area of TA in tumour‐bearing mice (*n* = 4 per group). (C) Representative periodic acid–Schiff (PAS) staining of TA and GC muscles in C26 and control mice; graph quantification of PAS staining in TA muscles (*n* = 4 per group). (D) *In vivo* measurement of absolute muscle force of gastrocnemius muscle and graph quantification of tetanic force (100 Hz) in C26 tumour‐bearing and sham control mice (*n* = 45 muscles per group, taken from 23 animals). (E) Normalized muscle force and tetanic quantification reveal a significant reduction in muscle function in cachectic mice (*n* = 45 per group, *n* = 23 mice). (F) Western blot analysis of mTOR signalling and related quantification of 4E‐BP1 and S6 reveal a decrease in mTORC1 activity (*n* = 9 per group). (G) Western blot and quantification of puromycin in C26 and sham mice. Data presented as mean ± SEM. Unpaired two‐tailed Student's *t*‐test. *P < 0.05, **P < 0.01, and ^****^ *P* < 0.0001.

Numerous types of cancers are associated with a loss in body weight due to a reduction in muscle and adipose tissue. To understand the underlying causes for this muscle wasting, we examined signalling and functional changes in murine models for cancer cachexia. The most used models are those that perform a subcutaneous implantation of cells for either C26 or LLC. As can be seen in *Figures* 1A and S2A, both experimental models lead to the expected loss in muscle weight and adipose tissue. Particularly, the C26 model shows a very clear and reproducible reduction in food intake (*Figure* S2B) and body weight, starting around Day 10 after inoculation. To obtain more insight into the molecular mechanisms underlying this muscle wasting, we analysed muscles in the C26 model after mice lost around 20% of initial body weight. Basic histological analysis revealed muscle atrophy but not changes in mitochondrial distribution in cachectic mice (*Figure* 1B). We did however observe a reduction in glycogen content in the TA muscle of tumour‐bearing mice (*Figure* 1C). Next, we examined muscle force production *in vivo*, by measuring torque production after electrical stimulation of the sciatic nerve. As can be observed in *Figure* 1D and 1E, not only we did observe a reduced absolute force production due to atrophy, but also when force is normalized for muscle mass, a small, yet significant reduction in maximal tension persists. One of the major regulators of muscle mass and force is the Akt–mTORC1 pathway.^3^ To determine if the observed loss of muscle mass and force is accompanied by alterations in this signalling pathway, we performed a western blotting analysis. As can be seen in *Figures* 1F, S2C, and S2D, we observed a general decrease in the activation of this pathway, with a trend towards a reduction in the phosphorylation of Akt and ribosomal protein S6 (S6). The total levels of S6 and eukaryotic translation initiation factor 4E binding protein 1 (4E‐BP1) are significantly altered in the C26 cachexia model. Next, we examined if this general down‐regulation of mTORC1 signalling is also accompanied by changes in protein synthesis rates. As can be seen in *Figure* 1G, the reduction in mTORC1 signalling is also accompanied by a decrease of 57% in puromycin incorporation in tumour‐bearing animals.

### Loss of mTORC1 during cachexia increases autophagic flux

**Figure 2:**
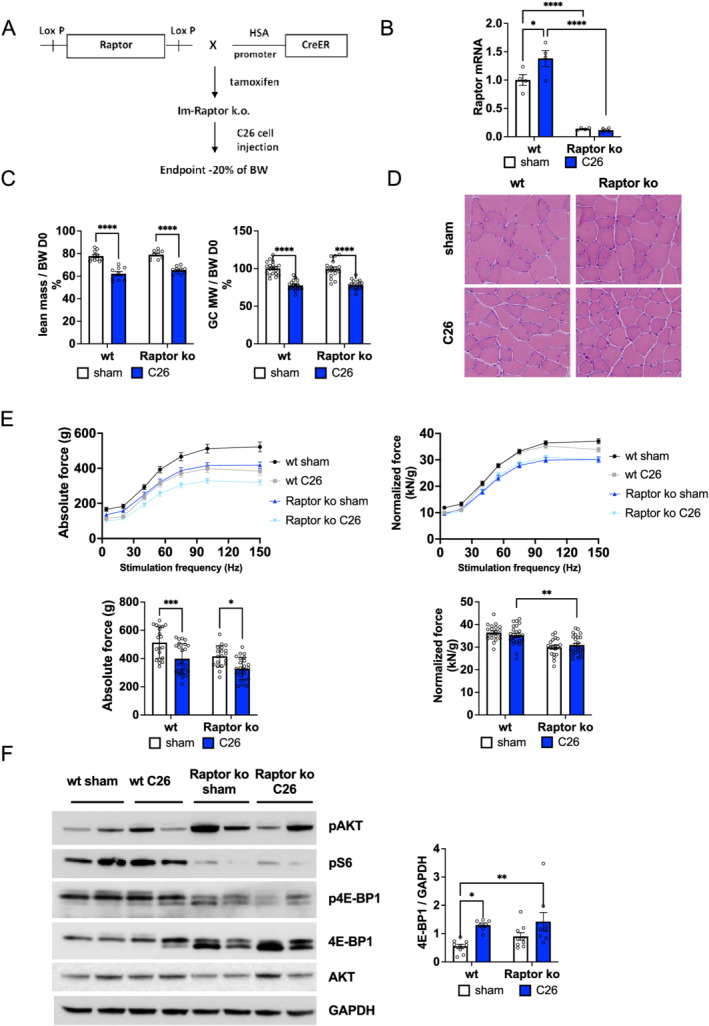
Characterization of tumour‐bearing Raptor ko mice. (A) Schematic representation of the experimental procedure: after inducible muscle‐specific deletion of Raptor gene through tamoxifen treatment, C26 cells were injected subcutaneously on the back of mice. Mice were sacrificed when they lose about 20% of body weight, generally 14 days after C26 cell inoculation. (B) Gene expression of Raptor in GC muscles for Raptor ko mice during cancer cachexia; data shown as expression relative to wt sham (Raptor gene: *n* = 4 per group). (C) Final lean mass, measured as total body mass minus fat mass, and gastrocnemius weight in Raptor ko tumour‐bearing mice do not change with respect to wt C26 mice (lean mass Raptor ko: *n* = 10 per group; GC weight Raptor ko: *n* = 18 per group). (D) Representative H&E staining of TA muscle in C26 Raptor ko mice and controls. (E) Absolute and normalized muscle force of GC muscle in Raptor ko C26 and sham mice and histogram of tetanic force at 100 Hz (*n* = 20 muscles, *n* = 10 mice for wt and Raptor ko sham; *n* = 24 muscles, *n* = 12 mice for wt and Raptor ko C26). (F) Representative western blot analysis of mTOR signalling in Raptor ko C26 mice and 4E‐BP1 quantification in Raptor ko C26 and sham mice (4E‐BP1: *n* = 9 per group). Data presented as mean ± SEM. Two‐way ANOVA with Tukey's multiple comparison *post hoc* test was performed. *P < 0.05, **P < 0.01, ^***^ *P* < 0.001, and ^****^ *P* < 0.0001.

**Figure 3:**
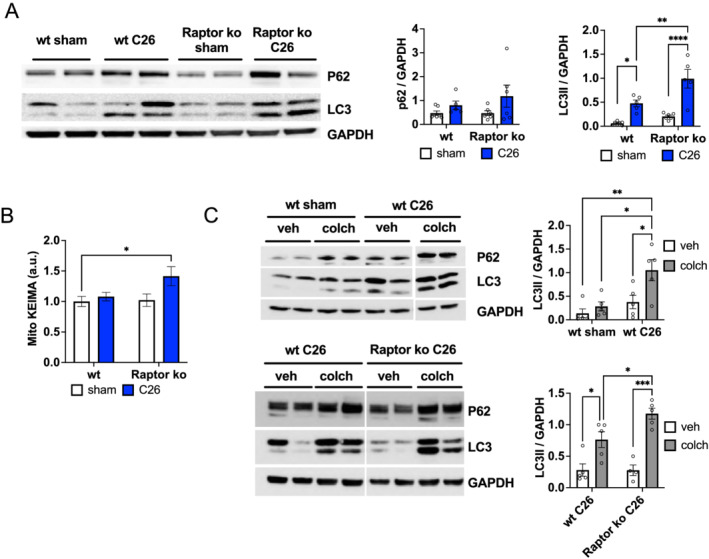
Raptor deletion leads to an increase in autophagic flux during cancer cachexia. (A) Protein expression of autophagy markers, LC3 and p62, in wt and Raptor ko, sham and C26 animals (*n* = 7 wt sham, *n* = 5 wt C26, *n* = 6 Raptor ko sham, and *n* = 5 Raptor ko C26). (B) Mito‐Keima measurement shows an increase in skeletal muscle mitophagy in Raptor ko C26 mice (*n* = 4 muscles per group). (C) Analysis of autophagic flux with colchicine treatment in Raptor ko mice during cancer cachexia. Representative p62 and LC3 western blots: quantification of wt sham and C26 animals, treated with colchicine or vehicle, and of wt and Raptor ko mice, colchicine or vehicle, during cancer cachexia (*n* = 5 per group). Data presented as mean ± SEM. Two‐way ANOVA with Tukey's multiple comparison *post hoc* test was performed. *P < 0.05, **P < 0.01, ^***^ *P* < 0.001, and ^****^ *P* < 0.0001.

As numerous cancer treatments aim at reducing Akt–mTOR activity in the tumour, we wondered if this systemic reduction in Akt–mTORC1 signalling could have adverse effects on skeletal muscle during cancer cachexia, possibly by reducing mTORC1 signalling even further. Indeed, the use of mTOR inhibitors in the clinic has been linked to increased muscle wasting in patients.^15^ To address this, we used a transgenic mouse line, in which we can inducibly delete Raptor (mTORC1), specifically in skeletal muscle. We inoculated tumour cells 3 weeks after the end of tamoxifen treatment, a time point at which animals do not show a significant phenotype yet^24^ (*Figures* 2A, 2B, and S3A). As can be seen in *Figures* 2C, S3B, S3C, and S4A, mice lacking Raptor from skeletal muscle show the same loss in overall lean and fat mass, muscle mass, and fibre size after inoculation of C26 tumour cells. Also, with regard to muscle histology and muscle force, we observed the same fibre atrophy in wild‐type and transgenic animals during cancer cachexia (*Figure* 2D and 2E). When we performed an analysis of the signalling changes, we observed the expected decrease in S6 phosphorylation together with a strong increase in total 4E‐BP1 and phosphorylated Akt levels in Raptor ko animals (*Figures* 2F and S4B). Next, we evaluated the regulation of autophagy and the role played by mTORC1, as autophagy is known to be activated during cancer cachexia and very sensitive to changes in mTORC1 activity levels. When examining the lipidation of microtubule‐associated protein 1A/1B‐light chain 3 (LC3) and the scaffold protein p62 in wild‐type mice, we confirmed the previously reported increase in LC3 lipidation in cachexia, while p62 showed a trend to increase (*Figure* 3A). Interestingly, loss of Raptor leads to a slight increase in LC3 lipidation under normal conditions, as we observed previously,^7^ which is markedly increased during cancer cachexia. To understand if this increased LC3 lipidation is due to an increased autophagic flux or a block in autophagy, we measured mitophagic flux in isolated fibres using the mito‐Keima probe. As shown in *Figure* 3B, we observed an increase in mitophagic flux in Raptor ko mice during cachexia. Lastly, to determine bulk autophagic flux, we treated wild‐type and Raptor knockout mice with colchicine to inhibit the fusion of autophagic vesicles with the lysosome. Western blotting analyses in the different conditions revealed an increase in LC3 lipidation in cachectic muscles after colchicine treatment, showing that bulk autophagic flux is increased. Interestingly, tumour‐bearing Raptor ko mice show 1.5‐fold increase in LC3 lipidation after colchicine treatment, suggesting more pronounced induction of bulk autophagy after loss of mTORC1 than that observed in wild‐type muscles (*Figures* 3C and S4C).

### Activation of Akt is sufficient to revert loss in muscle mass and force

**Figure 4:**
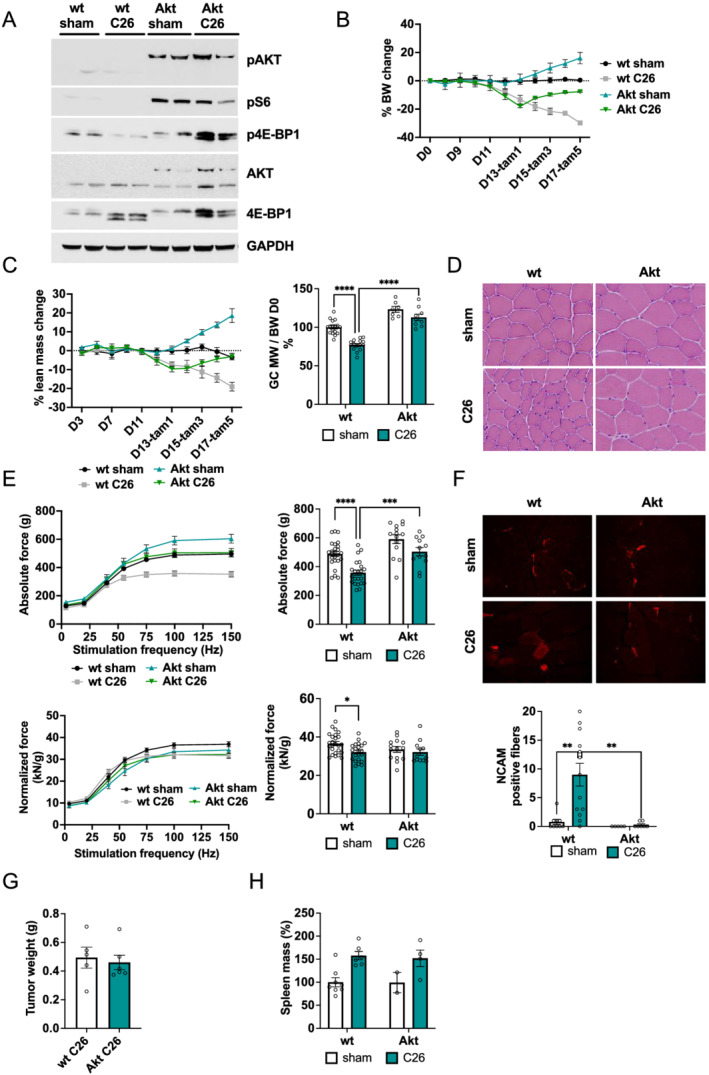
Activation of Akt–mTORC1 signalling restores body weight loss and muscle force during cancer cachexia. (A) Expression levels of phosphorylated AKT‐ER (Ser473); total AKT of endogenous AKT (55 kDa) and of myrAKT‐ER (75 kDa) in sham and tumour‐bearing Akt and wt mice; western blot analysis of mTOR signalling in Akt C26 mice and shams. (B) Representation of body weight progression in wt and Akt mice with or without inoculation of C26: when mice lose about 10–12% of body weight, tamoxifen is administered daily for 5 days to activate Akt. (C) Measurement of lean mass during cancer cachexia and its restoration after Akt activation; GC mass completely recovers in Akt C26 mice (*n* = 17 wt sham, *n* = 15 wt C26, *n* = 7 Akt sham, and *n* = 9 Akt C26). (D) Images of H&E staining in Akt C26 mice and controls. (E) Absolute and normalized muscle force *in vivo* in Akt C26 mice; quantification of tetanic force reveals a restoration in muscle force (*n* = 25 muscles, *n* = 13 mice wt sham; *n* = 22 muscles, *n* = 12 mice wt C26; *n* = 14 muscles, *n* = 12 mice Akt sham; and *n* = 12 muscles, *n* = 6 mice Akt C26). (F) Representative images of NCAM staining in GC muscle and related quantification of total number of positive fibres per muscle section in Akt C26 mice and controls. (G) Tumour weight in Akt and wt tumour‐bearing mice (*n* = 6 per group); unpaired two‐tailed Student's *t*‐test. (H) % spleen mass in Akt and wt tumour‐bearing mice; data shown as expression relative to wt sham (*n* = 8 wt sham, *n* = 6 wt C26, *n* = 2 Akt sham, and *n* = 6 Akt C26). Data presented as mean ± SEM. Two‐way ANOVA with Tukey's multiple comparison *post hoc* test was performed. *P < 0.05, **P < 0.01, ^***^ *P* < 0.001, and ^****^ *P* < 0.0001.

As cancer cachexia is associated with reduced Akt–mTORC1 signalling, we wondered if reactivation of this signalling pathway would be sufficient to counteract cancer‐related muscle wasting. To determine this, we used a mouse model in which we can inducibly activate Akt only in skeletal muscle. Briefly, a constitutively active, myristoylated form of Akt is expressed in skeletal muscle and fused to an ER domain, making it unstable leading to its rapid degradation. Administration of tamoxifen stabilizes and activates Akt, leading to a rapid muscle growth in healthy, normal muscle.^5^ To determine the effect of Akt activation on body weight and muscle wasting during cachexia, we implanted C26 cells and started administrating tamoxifen when mice had lost 10–15% of body weight. As can be seen in *Figure* 4A and 4B, muscles showed a very strong activation in Akt–mTORC1 signalling, and mice started gaining body weight almost immediately after starting tamoxifen treatment. Using EchoMRI to measure whole‐body lean mass, we find that activation of Akt for 5 days is sufficient to completely restore the loss in lean mass and muscle weight (*Figures* 4C and S5A). Analysis of muscle histology showed no pathological signs in C26 mice after Akt activation (*Figure* 4D), and glycogen depletion was prevented (*Figure* S5B). Next, we wondered if this increase in muscle mass after Akt activation is also accompanied by an increase in muscle force. As can be observed in *Figure* 4E, absolute muscle force in cachectic mice is completely restored after Akt activation. Furthermore, the reduction in normalized tetanic force observed in wild‐type cachectic mice is no longer observed after Akt activation, suggesting a complete rescue of muscle mass and function. As muscle force is determined after electrical stimulation through the sciatic nerve, we analysed the appearance of denervated fibres during cachexia. In line with an important role for mTORC1 signalling in maintaining muscle innervation, we did not observe the appearance of NCAM1‐positive fibres in cachectic mice after Akt activation (*Figure* 4F). Interestingly, we did not observe the appearance of more denervated fibres in Raptor ko mice during cachexia, as compared with sham ko animals (*Figure* S5C), possibly suggesting that part of the muscle denervation during cancer cachexia is due to reduced mTORC1 signalling. Importantly, this overall rescue of muscle wasting after Akt activation is not because it affects tumour weight or other systemic parameters, like increased spleen mass (*Figure* 4G and 4H).

### Activation of Akt normalizes the muscle transcriptome and reduces protein degradation

**Figure 5:**
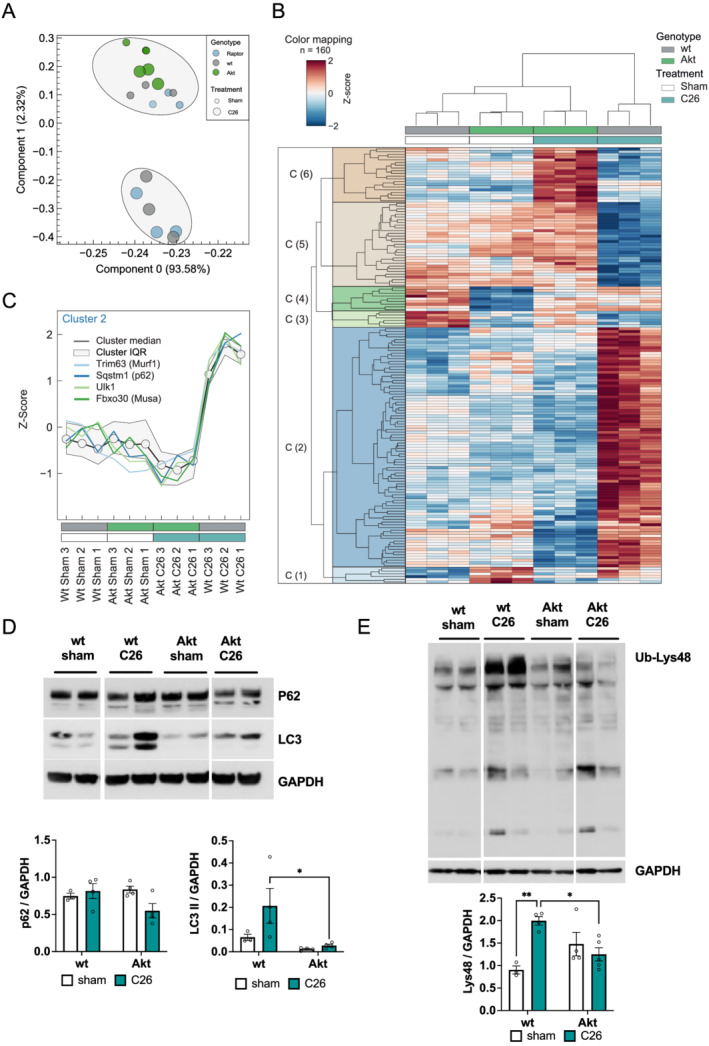
Activation of Akt recovers muscle transcriptome and protein degradation. (A) Principal component analysis of RNA‐Seq data of transgenic mice (Raptor ko, Akt, and wt) in control (sham) or post‐C26 inoculation (*n* = 3 animals per group). (B) Subset of genes after two‐way ANOVA comparing wt and Akt animals. Genes with interaction *P* value <0.001 are shown using Euclidean distance and the complete method. See also *Table* S1 for a heat map representation in Excel format. (C) Line plot visualizing Cluster 2 identified in (B) and genes of the proteasomal and autophagy system. IQR identifies the cluster inter‐quartile range. (D) Protein expression of autophagy markers, LC3 and p62, is restored after Akt activation during cancer cachexia (*n* = 3 wt sham, *n* = 4 wt C26, Akt sham, and Akt C26). (E) Western blot analysis of Lys48 ubiquitinated proteins and related quantification (*n* = 3 wt sham, *n* = 4 wt C26, Akt sham, and Akt C26). Data presented as mean ± SEM. Two‐way ANOVA with Tukey's multiple comparison *post hoc* test was performed. *P < 0.05 and **P < 0.01.

To determine the molecular mechanism behind this Akt‐dependent rescue of muscle wasting in an unbiased way, we performed RNA sequencing analysis of wild‐type, Raptor ko, and Akt overexpressing mice, both with and without inoculation of C26 cells. To obtain a systematic overview, we performed principal component analysis (*Figure* 5A). Under control conditions, the different transgenic mice did not segregate along the first two components. However, we observed that C26 cell inoculation induced distinct expression profiles in Raptor ko and wild‐type mice. Interestingly, activation of Akt in tumour‐bearing mice is sufficient to completely normalize the gene expression profile as seen by the principal component analysis. To identify statistically significant genes that are regulated during cachexia and behave differently between wt and Akt mice, we performed a two‐way ANOVA analysis (genotype and treatment). Next, we visualized the *z*‐score normalized expression values using a hierarchical clustering (*Figure* 5B) of genes (*n* = 160) that showed a significant interaction (*P* value <0.001). We detected several distinct clusters (*n* = 6, *Table* S1). Interestingly, by manual inspection of genes that are part of Cluster 2, we identified various genes, which are known to play an important role in protein degradation via the ubiquitin–proteasome [Trim63 (Murf1) and Fbxo30 (Musa)] or the autophagy–lysosome system [Sqstm1 (p62) and Ulk1] (*Figure* 5C). By RT‐qPCR, we confirmed the inhibitory effect of Akt on the induction of these genes in tumour‐bearing animals (*Figure* S6A). To investigate if transcriptional regulation results in altered activity of the degradative processes, we first evaluated levels of LC3 lipidation and total p62 abundance. We observed a strong reduction in LC3 lipidation and a trend for reduction in p62 after Akt activation, suggesting a reduction in autophagy in these muscles (*Figures* 5D and S6B). Next, we performed a western blotting analysis for lys48 ubiquitination to obtain an indication on the activity of ubiquitin–proteasome pathway. In line with the data of the RNA sequencing, we observed a complete blunting of protein ubiquitination in tumour‐bearing mice after Akt activation (*Figures* 5E and S6C).

Activation of Akt, however, does not only block increases in protein degradation but also the reduction in glycogen content in cachectic muscles is completely prevented by the activation of Akt. Lastly, the increase in the translational repressor 4EBP1 in muscles from tumour‐bearing animals, as shown in *Figure* 1, is completely prevented on both the mRNA and protein levels in Akt overexpressing animals (*Figure* S6D and S6E).

## Discussion

While cancer‐dependent muscle wasting is a well‐known problem in many, if not most, types of cancer, the underlying mechanisms are still not well described. This lack of mechanistic insight makes it very hard to anticipate when cachexia will start, how strong it will affect muscle physiology, and how it will influence the results of cancer treatment.

Here, we focused on one of the main regulators of adult skeletal muscle mass, the Akt–mTORC1 pathway, and how its activation levels affect skeletal muscle during cachexia. We find that loss of the core mTORC1 scaffold protein Raptor from skeletal muscle does not increase muscle wasting. It does, however, lead to an increase in autophagy during cachexia. Importantly, while loss of mTORC1 signalling does not lead to an increased muscle wasting, activation of Akt–mTORC1 in skeletal muscle is sufficient to completely revert the loss in mass and force in cachectic animals. This rescue is characterized by a general normalization of the transcriptome, completely blunting the induction of many atrophy‐related genes and reversing the increase in protein ubiquitination and autophagy seen in cachectic muscles.

Taken together, our data suggest that inhibition of mTOR during cachexia can negatively affect autophagy; however, its activation can completely restore muscle mass and function offering an interesting new therapeutic target for cancer cachexia.

### mTORC1 signalling affects autophagy and NMJ during cachexia

In this study, we evaluated if Akt–mTORC1 signalling is affected by cancer cachexia and if reducing it further has deleterious effects on muscle wasting. While not all studies observed a decrease in mTORC1 signalling during cachexia,^13^ our results are in line with the majority of reports showing a general decrease in mTORC1 signalling.^9^, ^11^ Some of the discrepancies observed in literature can be explained by the intrinsically high diurnal variability of this signalling pathway, making it very sensitive to aspects like food intake and intrinsic circadian timing,^25^ something not always easy to control for in cachectic animals. While we do not observe an increase in muscle wasting after loss of mTORC1 signalling, we do find an even further increase in autophagic flux. It is likely that the time frame of the muscle wasting (4–6 days) is too short to lead to major muscle defects, but it is tempting to assume that prolonging autophagy induction over time can eventually lead to a block in autophagy and the appearance of a muscle pathology, as reported previously.^7^ A similar observation was made in muscle‐specific Raptor ko mice after denervation. Indeed, also in this condition, autophagic flux was increased more than in wild‐type mice, leading to a more pronounced loss in muscle mass, which was most evident after 28 days.^6^ The increase in autophagic flux in Raptor ko mice during cachexia can be interpreted in two ways: (i) the induction of autophagy during cachexia is independent of mTORC1, and (ii) the reduction in mTORC1 is responsible for the induction of autophagy during cachexia, but the more pronounced reduction in mTORC1 signalling after genetic loss of function leads to an even further increase in autophagic flux through the same signalling pathway. While we cannot give a definite answer, it has been shown that knockdown of Raptor reduces signalling to downstream mediators; however, it does not eliminate their sensitivity to nutrient levels,^26^ leaving open the possibility that this increased autophagic flux during cancer cachexia is mTORC1 independent.

A newly described feature of cancer‐dependent muscle wasting is the fact that cachexia is accompanied by the appearance of fibre denervation.^8^ In this study, the authors show that reduced BMP signalling during cancer cachexia is a major factor leading to fibre denervation, as treatment with the BMP activator tilorone is sufficient to reduce muscle wasting and NMJ dysfunction. Interestingly, we and others have recently shown that one of the main roles of mTORC1 signalling in muscle fibres is the maintenance of the NMJ.^7^, ^27^ Considering that a significant part of the trophic effect of BMP signalling in skeletal muscle goes through its regulation of mTORC1 signalling,^28^ it is tempting to assume that also during cancer cachexia, the loss in fibre innervation is partially through a reduction in mTORC1 signalling in muscle fibres. Indeed, we do not find any further increase in denervated fibres in tumour‐bearing Raptor ko mice, and, importantly, we do not see the increase in fibre denervation in tumour‐bearing mice after Akt activation.

### Activation of Akt–mTORC1 signalling completely reverts cancer cachexia

The major finding of this study is the fact that activation of Akt for a few days is sufficient to completely restore muscle mass and force in already cachectic animals. This finding is of great importance, as it has been shown that the rescue of muscle mass is sufficient to improve survival, without affecting tumour growth.^29^ Modulating the levels of certain cytokines has been shown to be beneficial for muscle growth during cachexia. Antibodies that reduce activin/myostatin,^29^ FN14,^30^ or GDF15^31^ in the circulation have either prevented or reversed the muscle wasting seen during cachexia. While these results have shown the importance of preventing/reversing cachexia on overall survival, the intracellular signalling pathways involved are not well defined. Interestingly, activin, FN14, and GDF15 are all known to reduce the activation of the Akt–mTORC1 pathway,^29^, ^30^, ^32^ suggesting that preventing this inhibition is an important aspect of improving muscle wasting. Indeed, our results clearly show that muscle wasting can be completely reversed by short‐term activation of Akt signalling. Somewhat unexpected, this reversal of cachexia is also accompanied by a very robust alteration of the muscle transcriptome. As observed also by others, we find more up‐regulated genes than down regulated genes in cachectic muscles,^33^, ^34^ without major differences between tumour‐bearing wild‐type and Raptor ko mice. An important part of these up‐regulated genes code for proteins involved in protein degradation. Interestingly, activation of Akt completely abrogates the up‐regulation of most of these genes. This block in transcription of genes linked to protein degradation can be partially due to the strong inhibitory effect of Akt on FoxO transcription factors.^35^ FoxO transcription factors regulate an extensive network of genes linked to both the ubiquitin–proteasome and autophagy–lysosome system.^36^ Another support for a reduced activity of FoxO after Akt activation is the fact that the increase in the translational repressor 4E‐BP1 during cachexia is completely blocked by Akt activation, giving mechanistic insight to the muscle growth observed in these animals. This important role for FoxO‐dependent transcription during cancer cachexia was also suggested by studies overexpressing a dominant‐negative plasmid for FoxO.^34^ Indeed, this overexpression leads to a complete block in muscle atrophy and loss in muscle force in tumour‐bearing mice. The fact that we do not find a major change in transcriptome after Akt activation is in line with previous results^4^ and again suggests that Akt is able to prevent the pathological increase in transcription, which accompanies the progression of cancer cachexia. Also, evaluation of the expression of other FoxO target genes confirms the inhibition of FoxO activity after Akt activation in cachectic muscles (*Figure* S6F). It is tempting to speculate that also in more severe situations, like during chemotherapy or refractory cachexia, Akt maintains the capacity to stimulate muscle growth. It has been suggested that cachectic patients maintain anabolic capacity,^37^, ^38^ and we have previously shown that also in other pathological conditions, like muscular dystrophy or ageing,^19^, ^39^ activation of Akt is able to stimulate muscle growth. While it is not straightforward to imagine a systemic drug delivery that activates Akt only in skeletal muscles, the activation of muscle Akt can be considered an important marker in predicting the efficiency of nutritional and exercise interventions.

Taken together, we show in this manuscript that Akt–mTORC1 signalling plays an important role during cancer‐dependent muscle wasting. Loss of mTORC1 signalling is sufficient to further increase autophagic flux, which can potentially over time lead to debilitating effects on muscle mass and function. On the contrary, activation of this pathway in skeletal muscle is sufficient to completely rescue muscle wasting, creating interesting new therapeutic avenues for this debilitating aspect of many different cancers.

## Funding

This work was supported by grants from the French Muscular Dystrophy Association [Association Française contre les Myopathies (AFMTéléthon) to B.B., No. 21865], University of Padua (Università degli Studi di Padova) (STARS‐MyoAktivation to B.B.), and Associazione Italiana per la Ricerca sul Cancro (AIRC) 20406 to B.B.

## Conflict of interest

Alessia Geremia, Roberta Sartori, Martina Baraldo, Leonardo Nogara, Valeria Balmaceda, Georgia Ana Dumitras, Stefano Ciciliot, Marco Scalabrin, Hendrik Nolte, and Bert Blaauw declare that they have no conflict of interest.

## Supporting information


**Figure S1.** Treatment schemes transgenic animals.
Timeline representation of mice treatments for Raptor ko C26 mice and Akt C26 mice.
**Figure S2.** Muscle weight, body weight, and signaling changes in cancer cachexia models.
A. Quantification of body weight and gastrocnemius weight in LLC and sham mice (*n* = 5 wt sham, *n* = 8 wt LLC). B. Food intake progression in C26 and sham mice. (n = 8 wt sham, *n* = 10 wt C26). C. Quantification of western blot analysis in C26 mice (*n* = 9 per group). D. Western blot of mTOR signaling markers in LLC mice and controls and related quantification (*n* = 4 wt sham, *n* = 5 wt LLC). Data presented as mean ± s.e.m. Unpaired two‐tailed student's *t* test. ****p < 0.0001.
**Figure S3.** Skeletal muscle analysis of tumor‐bearing Raptor ko mice.
A. Representative Raptor western blot for Raptor ko tumor‐bearing mice and controls. B. Body weight progression during cancer cachexia in Raptor ko animals. *C*. Tibialis Anterior weight in Raptor ko tumor‐bearing mice (TA weight Raptor ko: *n* = 18 per group). Final fat mass measured by EchoMRI in Raptor ko C26 mice and related controls (fat mass Raptor ko: *n* = 10 per group). Two‐way ANOVA with Tuckey's multiple comparison post‐hoc test was performed. *p < 0.05, **p < 0.01, ***p < 0.001, ****p < 0.0001.
**Figure S4.** Changes in mTOR signaling and CSA in Raptor ko mice.
A. Cross sectional area of TA in Raptor ko C26 mice and controls (*n* = 4 wt sham, wt C26 and Raptor ko C26; *n* = 3 Raptor ko sham). Two‐way ANOVA with Newman–Keuls multiple comparisons test. B. Quantification of western blot of Fig 2F in Raptor ko C26 mice and controls (*n* = 9 wt sham, *n* = 7 wt C26, n = 9 Raptor ko sham, *n* = 8 Raptor ko C26). C. Uncut version of western blots in Fig 3C. Two‐way ANOVA with Tuckey's multiple comparison post‐hoc test was performed. Data presented as mean ± s.e.m. *p < 0.05, **p < 0.01, ***p < 0.001, ****p < 0.0001.
**Figure S5.** Muscle weight and histology in Akt mice, and NCAM‐positive fibers in Raptor ko mice.
*A*. Tibialis anterior weight recovers to wt sham level in Akt C26 mice (*n* = 8 wt sham, *n* = 6 wt C26 and Akt C26, *n* = 2 Akt sham). B Representative images and graph quantification of PAS staining in TA Akt C26 mice and controls. C. Quantification of NCAM positive fibers in Raptor ko C26 mice (expressed as percentage of NCAM‐positive vs total fiber number per section). Data presented as mean ± s.e.m. Two‐way ANOVA with Tuckey's multiple comparison test was performed. *p < 0.05, **p < 0.01, ***p < 0.001, ****p < 0.0001.
**Figure S6.** Akt activation reverts muscle transcriptome during cancer cachexia.
A. RT‐qPCR analysis of genes for ubiquitin‐proteasome (Trim63 (Murf1), Fbxo30 (Musa)) and autophagy‐lysosome system (Sqstm1 (p62), Ulk1) in Akt C26 mice (*n* = 3 wt sham, wt C26, Akt sham, *n* = 4 Akt C26 for Trim63, Ulk1 and Sqstm1; *n* = 5 wt sham, *n* = 6 wt C26, n = 4 Akt sham, *n* = 7 Akt C26 for Fboxo30). B. Uncut version of western blot in Figure 5D. C. Uncut version of western blot in Figure 5E. D. 4E‐BP1 expression in RNAseq analysis (*n* = 3 per group) and (E) protein level in Akt C26 mice (*n* = 4 wt sham, wt C26, Akt sham; *n* = 5 Akt C26). F. RT‐qPCR of FoxO‐dependent genes: Bnip3, Fbxo32 (Atrogin‐1) and Map 1lc3b (LC3) in Akt C26 mice (*n* = 3 wt sham, wt C26, Akt sham, *n* = 4 Akt C26). Data presented as mean ± s.e.m. Two‐way ANOVA with Tuckey's multiple comparison test was performed. *p < 0.05, **p < 0.01, ***p < 0.001, ****p < 0.0001.

**Table S1.** RNAseq results contain expression values as well as statistical tests (pairwise comparison and two‐way ANOVA results). A description of the column headers is available as well as a representation of the hierarchical clustering shown in Figure 5b.
