# Supplementary material for: Activation of Akt–mTORC1 signalling reverts cancer‐dependent muscle wasting
Source: J Cachexia Sarcopenia Muscle. 2021 Nov 6;13(1):648–61. doi: 10.1002/jcsm.12854 (PMC8818597; doi:10.1002/jcsm.12854)

Supplementary figure 1

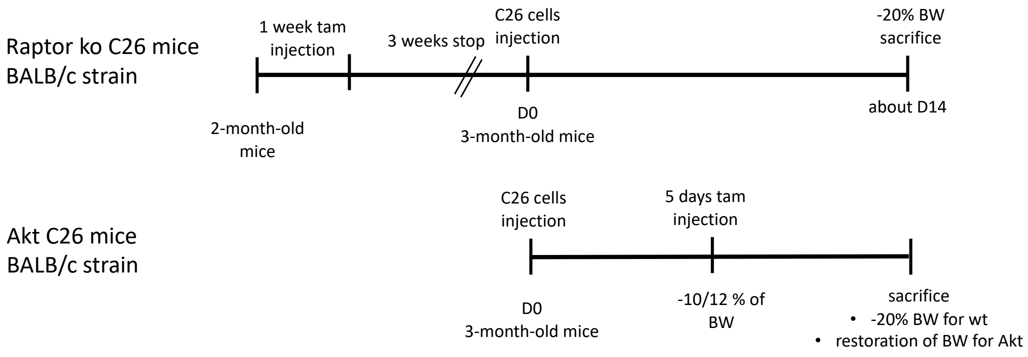

Supplementary figure 2

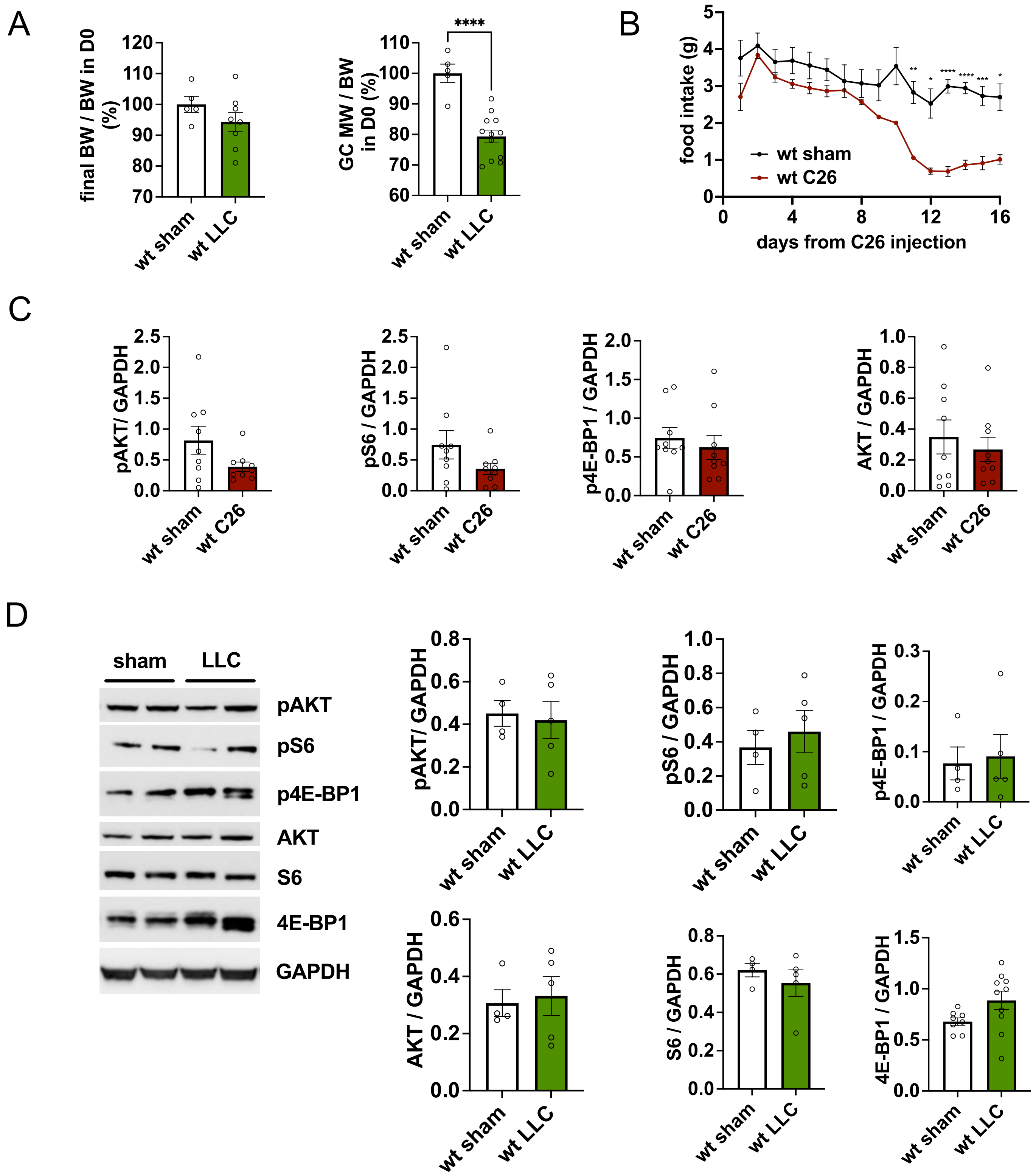

Supplementary figure 3

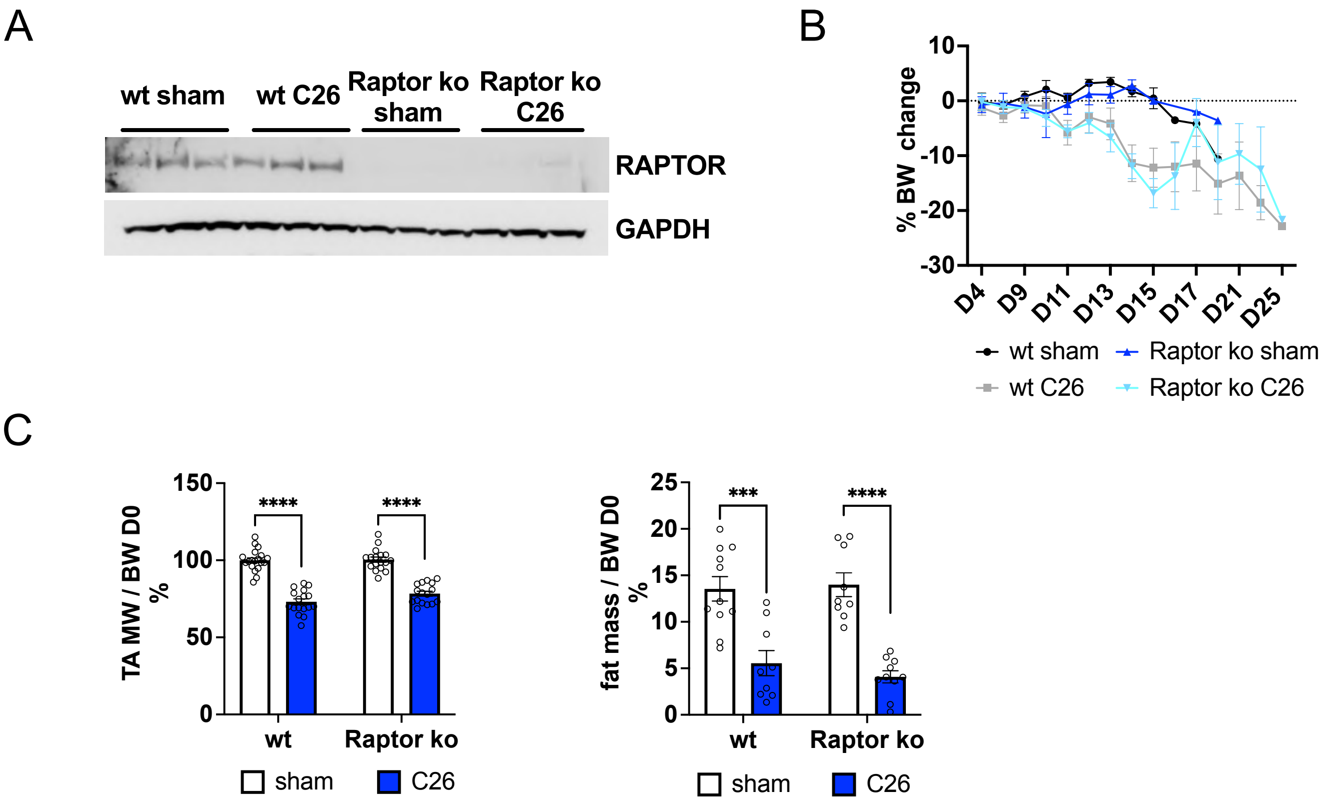

Supplementary figure 4

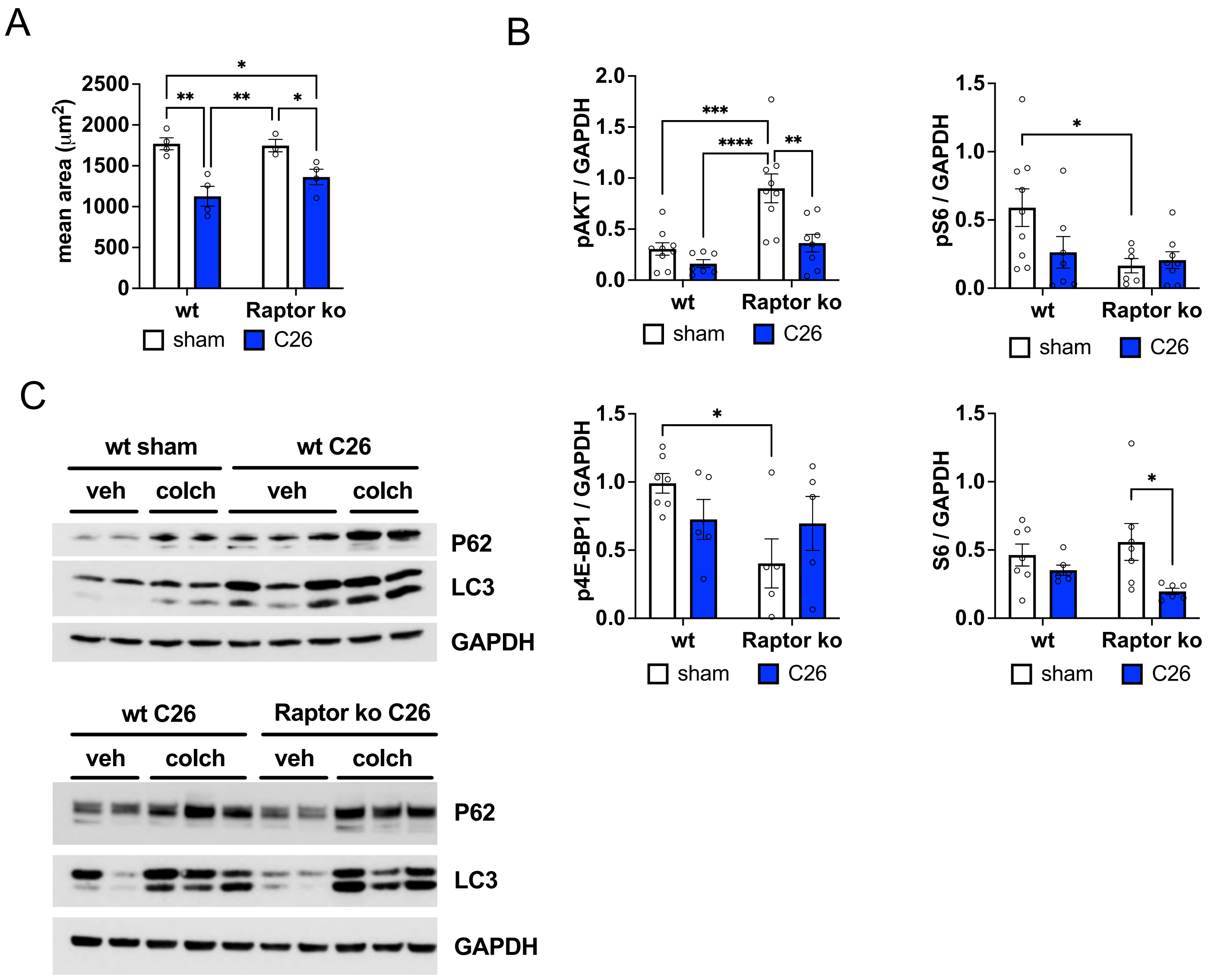

Supplementary figure 5

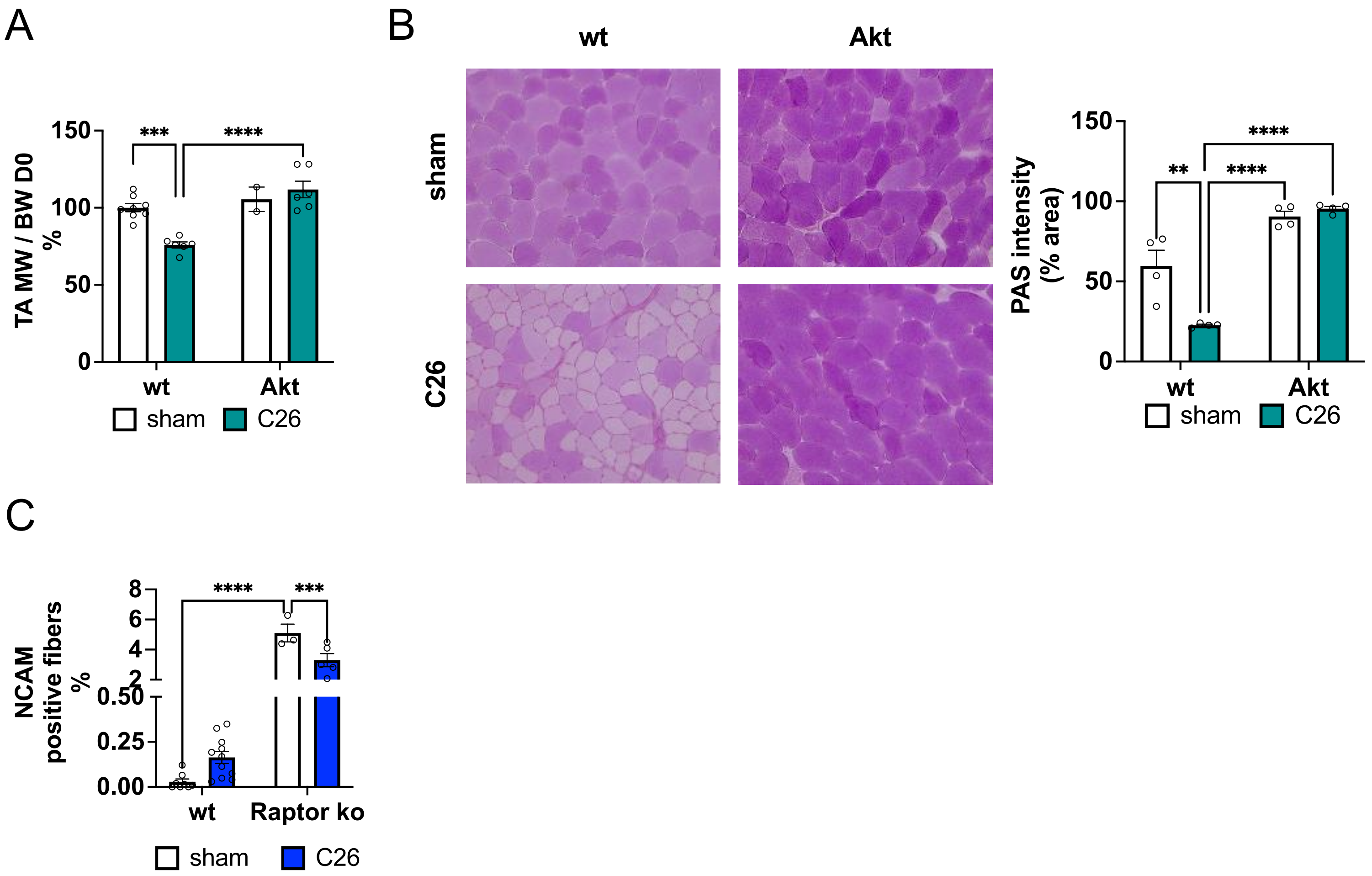

Supplementary figure 6

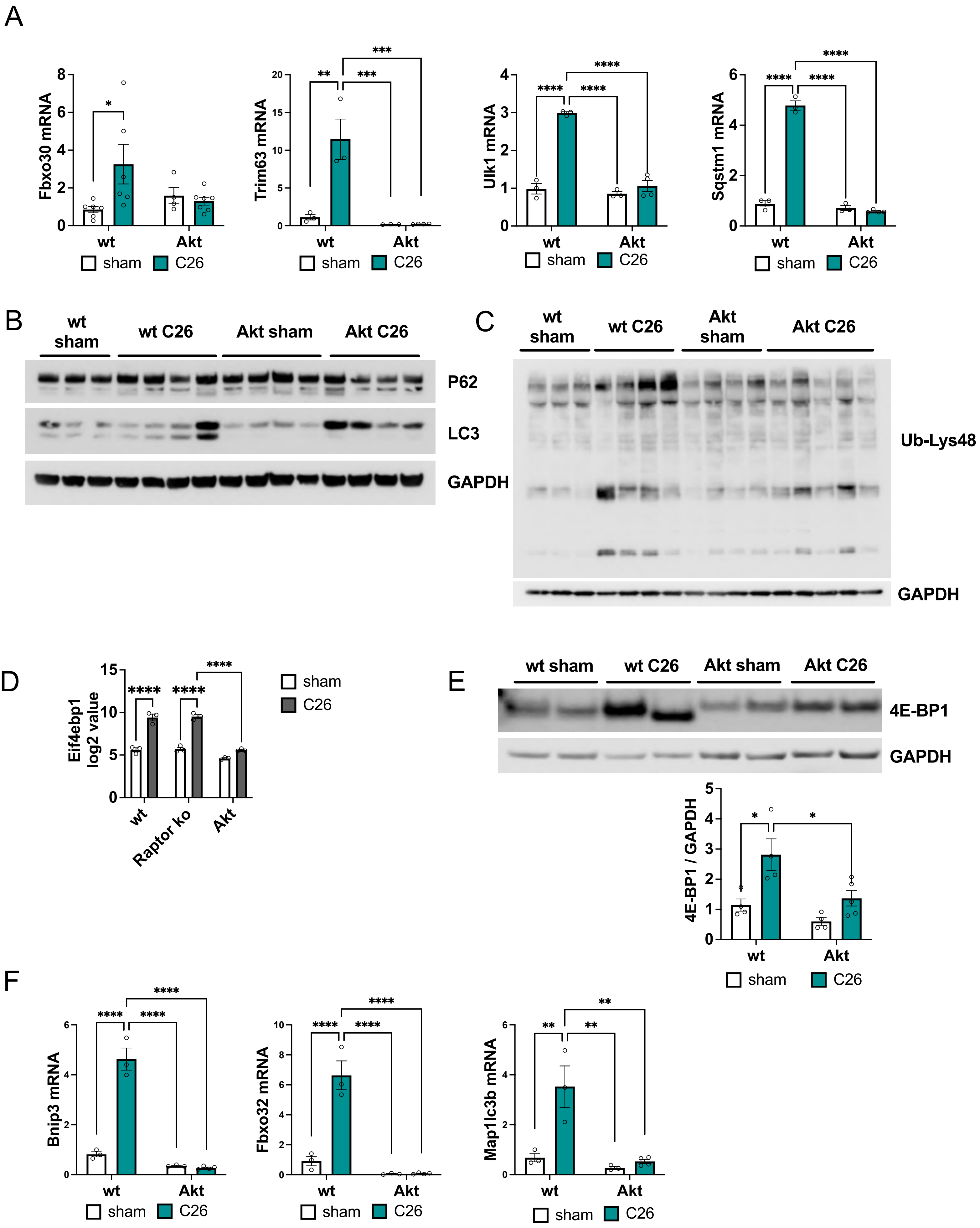

Supplement: Supplementary file 1 — Figure S1. Treatment schemes transgenic animals. Timeline representation of mice treatments for Raptor ko C26 mice and Akt C26 mice. Figure S2. Muscle weight, body weight, and signaling changes in cancer cachexia models. A. Quantification of body weight and gastrocnemius weight in LLC and sham mice (n = 5 wt sham, n = 8 wt LLC). B. Food intake progression in C26 and sham mice. (n = 8 wt sham, n = 10 wt C26). C. Quantification of western blot analysis in C26 mice (n = 9 per group). D. Western blot of mTOR signaling markers in LLC mice and controls and related quantification (n = 4 wt sham, n = 5 wt LLC). Data presented as mean ± s.e.m. Unpaired two‐tailed student's t test. ****p < 0.0001. Figure S3. Skeletal muscle analysis of tumor‐bearing Raptor ko mice. A. Representative Raptor western blot for Raptor ko tumor‐bearing mice and controls. B. Body weight progression during cancer cachexia in Raptor ko animals. C. Tibialis Anterior weight in Raptor ko tumor‐bearing mice (TA weight Raptor ko: n = 18 per group). Final fat mass measured by EchoMRI in Raptor ko C26 mice and related controls (fat mass Raptor ko: n = 10 per group). Two‐way ANOVA with Tuckey's multiple comparison post‐hoc test was performed. *p < 0.05, **p < 0.01, ***p < 0.001, ****p < 0.0001. Figure S4. Changes in mTOR signaling and CSA in Raptor ko mice. A. Cross sectional area of TA in Raptor ko C26 mice and controls (n = 4 wt sham, wt C26 and Raptor ko C26; n = 3 Raptor ko sham). Two‐way ANOVA with Newman–Keuls multiple comparisons test. B. Quantification of western blot of Fig 2F in Raptor ko C26 mice and controls (n = 9 wt sham, n = 7 wt C26, n = 9 Raptor ko sham, n = 8 Raptor ko C26). C. Uncut version of western blots in Fig 3C. Two‐way ANOVA with Tuckey's multiple comparison post‐hoc test was performed. Data presented as mean ± s.e.m. *p < 0.05, **p < 0.01, ***p < 0.001, ****p < 0.0001. Figure S5. Muscle weight and histology in Akt mice, and NCAM‐positive fibers in Raptor ko mice. A. Tibialis anterio [file JCSM-13-648-s001.pdf]
